# Supplementary material for: Electrochemomechanical Behavior of Polypyrrole-Coated Nanofiber Scaffolds in Cell Culture Medium
Source: Polymers (Basel). 2019 Jun 13;11(6):1043. doi: 10.3390/polym11061043 (PMC6630290; doi:10.3390/polym11061043)
Supplement: Supplementary file 1 [file polymers-11-01043-s001.pdf]

# Supplementary

## Electrochemomechanical behavior of polypyrrole-coated nanofiber scaffolds in cell culture medium

Madis Harjo[1], Janno Torop[1], Martin Järvekülg[2], Tarmo Tamm[1] and Rudolf Kiefer[3,*]

[1]Intelligent Materials and Systems Lab, Faculty of Science and Technology, University of Tartu, Nooruse 1, 50411 Tartu, Estonia

[2]Institute of Physics, Faculty of Science and Technology, University of Tartu, W. Ostwaldi Str 1, 50411 Tartu, Estonia

[3]Conducting polymers in composites and applications Research Group, Faculty of Applied Sciences, Ton Duc Thang University, Ho Chi Minh City, Vietnam

*Corresponding author. Tel: +886 905605515. E-mail: rudolf.kiefer@tdtu.edu.vn (Rudolf Kiefer)

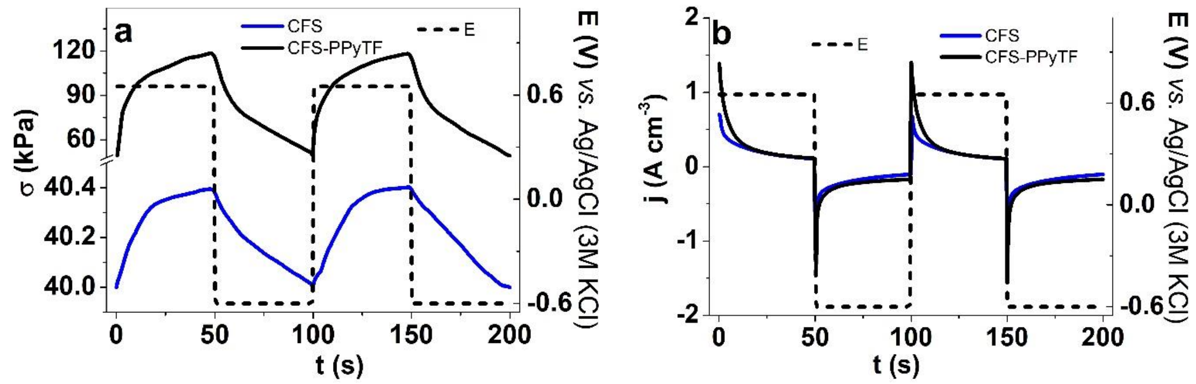


Figure S1. Square wave potential waves at 0.65 V to-0.6 V in CCM solution of CFS (blue) and CFS-PPyTF samples (black) at 0.01 Hz showing in a: the stress σ and in b: the current density time cycles of two subsequent cycles (3rd and 4th) against the time t.

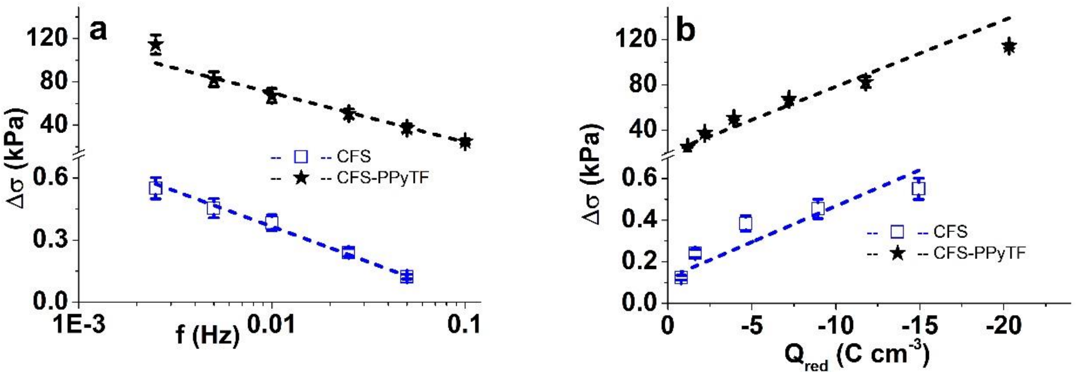


Figure S2. Square wave potential steps at applied frequencies 0.0025 Hz to 0.1 Hz in CCM solution at applied voltage 0.65 to -0.6 V of CFS samples (blue, □) and CFS-PPyTF samples (black, ★) showing a: the stress difference Δσ against applied frequencies f (logarithmic scale) and in b: the stress difference Δσ against charge density at reduction $Q_{red}$. The dashed line are shown only for orientation and representing the linear fit (y = a + b*x, with adj. R square ($R^2$) of 0.97 for CFS-PPyTF and 0.99 for CFS).

Table S1. Strain ε and stress differences Δσ of CFS and CFS-PPyTF at potential range 0.65 V to -0.6 V in mean values with standard deviations

| Samples | 0.0025 Hz | 0.005 Hz | 0.01 Hz | 0.025 Hz | 0.05 Hz | 0.1 Hz |
|---|---|---|---|---|---|---|
| CFS, ε [%] | 0.27 ±0.02 | 0.23 ± 0.02 | 0.2 ±0.01 | 0.13 ± 0.01 | 0.1 ±0.01 | - |
| CFS, Δσ [kPa] | 0.55 ±0.05 | 0.45 ± 0.04 | 0.38 ±0.04 | 0.24 ± 0.02 | 0.12 ± 0.01 | - |
| CFS-PPyTF, ε [%] | 0.88 ±0.07 | 0.64 ± 0.05 | 0.5 ±0.05 | 0.38±0.04 | 0.3 ±0.02 | 0.17 ± 0.02 |
| CFS-PPyTF, Δσ [kPa] | 114.4 ±8.9 | 82.5 ±7.6 | 67.3 ±6.5 | 50.5 ±5.5 | 37.2 ±3.8 | 24.8 ± 2.3 |
